# Caveolin-1 interacts with the Gag precursor of murine leukaemia virus and modulates virus production

**DOI:** 10.1186/1743-422X-3-73

**Published:** 2006-09-06

**Authors:** Zheng Yu, Christiane Beer, Mario Koester, Manfred Wirth

**Affiliations:** 1 Molecular Biotechnology Division, German Research Centre for Biotechnology, GBF, Mascheroder Weg 1, Braunschweig, Germany; 2 Department of Molecular Biology, Aarhus University, C.F. Mollers Alle 130, Aarhus, Denmark

## Abstract

**Background:**

Retroviral Gag determines virus assembly at the plasma membrane and the formation of virus-like particles in intracellular multivesicular bodies. Thereby, retroviruses exploit by interaction with cellular partners the cellular machineries for vesicular transport in various ways.

**Results:**

The retroviral Gag precursor protein drives assembly of murine leukaemia viruses (MLV) at the plasma membrane (PM) and the formation of virus like particles in multivesicular bodies (MVBs). In our study we show that caveolin-1 (Cav-1), a multifunctional membrane-associated protein, co-localizes with Gag in a punctate pattern at the PM of infected NIH 3T3 cells. We provide evidence that Cav-1 interacts with the matrix protein (MA) of the Gag precursor. This interaction is mediated by a Cav-1 binding domain (CBD) within the N-terminus of MA. Interestingly, the CBD motif identified within MA is highly conserved among most other γ-retroviruses. Furthermore, Cav-1 is incorporated into MLV released from NIH 3T3 cells. Overexpression of a GFP fusion protein containing the putative CBD of the retroviral MA resulted in a considerable decrease in production of infectious retrovirus. Moreover, expression of a dominant-negative Cav-1 mutant affected retroviral titres significantly.

**Conclusion:**

This study demonstrates that Cav-1 interacts with MLV Gag, co-localizes with Gag at the PM and affects the production of infectious virus. The results strongly suggest a role for Cav-1 in the process of virus assembly.

## Background

The Gag protein precursor is a polyprotein consisting of matrix protein (MA), protein p12, capsid protein (CA) and nucleocapsid protein (NC) and represents a principal actor in retrovirus assembly at the plasma membrane (PM). The Gag precursor is synthesized on free ribosomes and myristoylated at glycin 2 in MA. Fatty acylation is sufficient to localize Gag at the plasma membrane, where in the presence of the envelope (Env) proteins viral particles assemble. In the final stage of budding a membrane fission event is required for efficient separation of newly synthesized retroviruses. Concurrent with budding, the Gag polyprotein is cleaved by the retroviral protease into MA, CA, NC and other virus-specific Gag derived proteins. Distinct regions in the Gag protein were identified which mediate membrane binding, multimerization and induce separation of nascent virus particles from the cell [1].

Gag alone is sufficient to induce the formation of virus-like particles (VLPs) [1]. The formation of infectious particles, however, requires co-localization of Env and Gag and occurs in a cell-dependent manner either at the plasma membrane or at internal membranes. Mutational analysis of Gag of certain retroviruses defined several regions important for Gag transport and efficient membrane anchoring, as mutant viruses were blocked in membrane association or redirected to multivesicular bodies (MVBs) localized in the cytoplasm. It has been shown, that myristoylation of Pr65^gag ^at Gly2 at the aminoterminus of the viral MA is substantial for Moloney murine leukaemia virus (MoMLV) particle formation and budding [2,3], and is required for efficient binding to the plasma membrane. In addition, a run of basic residues or a cluster of lipophilic amino acids close to the aminoterminus are involved in Gag transport to the site of virus assembly [4-8] However, the requirement for fatty acylation can be overcome by other molecules. Thus, protein-protein interactions have been postulated to be necessary for efficient protein localization in lipid rafts [9].

Surprisingly, Gag transport turned out to be a complex process involving several cellular proteins. Early experiments with MLV and Rous sarcoma virus (RSV) demonstrated that deletion of a region located between MA and CA affected virus assembly. The same was true for human immunodeficiency virus (HIV) when a region at the carboxy-terminus of the Gag precursor was deleted. These early notions led to the identification of L-domains which recruit the cellular machinery for intravesicular transport of Gag [reviewed in 10,11]. The identified L-domains differ in their sequence within the retrovirus family and each retroviral L-domain binds specific factors to redirect Gag into the MVB pathway, thereby directing the budding and egress of virions. The subject is still puzzling, as viral Gag proteins contain several interacting motifs and their importance varies in different retroviruses [12].

Formation of infectious MLV as well as successful pseudotyping of MLV vectors requires Env co-colocalization with Gag [13,14]. Moreover, incorporation of cellular membrane proteins into virions seems to be dependent on co-localization. Interestingly, the incorporation reflects the cell type, intracellular transport and the platform of assembly and budding. Lipid rafts have been suggested as portals for retrovirus exit. Env localization in lipid rafts has been demonstrated for ecotropic MoMLV [15] as well as for amphotropic 4070A MLV more recently [16].

When studying 4070A Env localization in infected NIH3T3 cells, we noticed co-localization of Env with Cav-1, a multi-functional membrane protein. Cav-1 is present in lipid rafts and its oligomerization leads to caveolae formation. Caveolae are the main actors for a clathrin-independent endocytic pathway, first identified for internalization of GPI anchored proteins [17]. Moreover, Cav-1 functions as scaffolding protein to organize and concentrate a growing list of proteins involved in diverse signaling processes. Finally, Cav-1 is involved in cholesterol transport [18].

We wondered whether the co-localization of Cav-1 and 4070A Env in the PM of mouse NIH3T3 cells results in release of 4070A MLV (referred to as A-MLV) containing Cav-1. Here, we proved the presence of considerable amounts of Cav-1 within A-MLV as well as MoMLV. Furthermore, MLV Gag co-localizes with Cav-1 at the PM. Co-immuno-precipitations revealed that both proteins interact presumably via a caveolin-binding domain (CBD) within the aminoterminal region of MA. The CBD is highly conserved in Gag of most γ-retroviruses and competition experiments using CBD fusion proteins or a Cav-1 dominant-negative mutant revealed that Gag-Cav-1 interaction modulates MLV production.

## Results

### Amphotropic and ecotropic murine leukemia virions incorporate Caveolin-1

**Figure 1:**
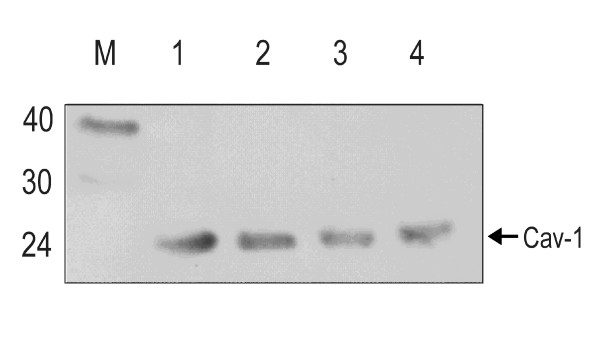
**Caveolin-1 is incorporated into MLV virions**. Ecotropic MLV and amphotropic MLV were pelleted from supernatants of infected NIH3T3 cells and purified by sucrose gradient centrifugation and virions were lysed and subjected to SDS-PAGE (10%) followed by Western blot analysis using rabbit anti Cav-1 as primary antibody. Lane 1: Cav-1 positive control, human carcinoma cell lysate; Lane 2: Cav-1 positive control, NIH 3T3 cell lysate. Lane3: Ecotropic MLV Lane 4: Amphotropic MLV. M Marker, molecular weight in kDa.

Recently, we reported co-localization of Cav-1 and Env of A-MLV [16] and presented hints for Cav-1 incorporation into A-MLV released from mouse NIH 3T3 cells [19]. To confirm our initial results and to extend our findings to the ecotropic MoMLV we investigated whether Cav-1 is included into released virions. For that purpose we analysed A-MLV and MoMLV propagated in NIH 3T3 cells. Viruses were purified by ultracentrifugation followed by sucrose gradient centrifugation. Viral proteins were separated by SDS-PAGE and analysed by Western Blot using anti-Cav-1 antibodies. Figure 1 demonstrates that Cav-1 is incorporated into MoMLV (lane 3) as well as A-MLV (lane 4). A signal with the size expected for Cav-1 isoforms (21–24kD) could be detected in processed virus samples (lane 3,4) which comigrates with positive control samples from cell lysates (lane 1,2). Processed supernatants of mock-infected non-virusproducing NIH 3T3 cells did not give rise to a signal (data not shown, [19]). Therefore, both MLV strains incorporate Cav-1 into the viral membrane, presumably during the process of budding from lipid rafts of NIH 3T3 cells.

### Co-localization of MLV-Gag and caveolin-1

**Figure 2:**
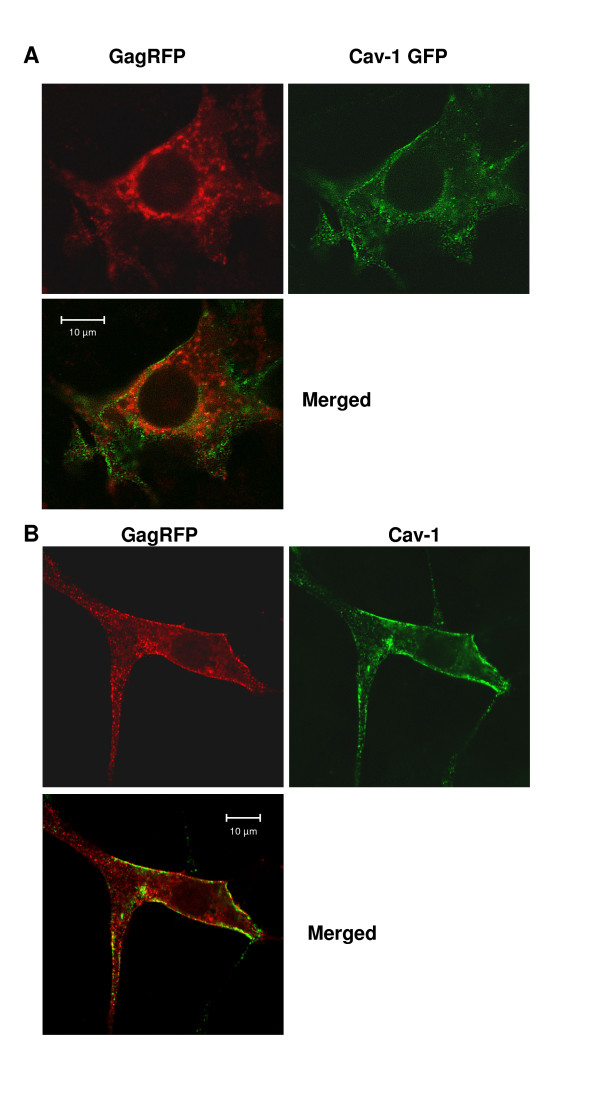
**Colocalization studies using confocal microscopy**. **A. **Caveolin-1-GFP and GagRFP fusion proteins colocalize in transiently transfected NIH 3T3 cells predominantly at the plasma membrane. NIH 3T3 cells were co-transfected with Gag-RFP and caveolin-1 GFP plasmids, fixed 46 h later and analysed by confocal microscopy. **B. **Caveolin-1 and GagRFP colocalization in NIH3T3 cells. NIH3T3 transfected with GagRFP plasmid were fixed 46 h after transfection and stained for immunofluorescence rabbit anti-caveolin-1antibody followed by goat anti-rabbit-Alexa 488 conjugate

To address the question whether MLV Gag and Cav-1 co-localize, we investigated NIH 3T3 and 293 cells transfected with expression plasmids carrying Gag or Cav-1 fused C-terminally to fluorescent proteins (FPs) using confocal microscopy. It has been shown that attachment of GFP to the C-terminus of Cav-1 does not interfere with its localization, fatty acylation or oligomerization properties [20-22]. Similarly, C-terminal fusions of FPs to Gag have been proven to be valuable tools to study localization, incorporation and budding of MLV Gag [23,24]. NIH 3T3 cell transfected with Cav-1-GFP, showed a typical surface staining and exhibited the expected expression pattern, but also several scattered spots distributed throughout the cytoplasm and a prominent accumulation of Cav-1 positive membranes at the center of the cell in perinuclear regions were observed (Fig. 2A). Gag-RFP appeared in a punctate pattern distributed allover the cytoplasm, accumulated in membranes in perinuclear regions and at or close to the PM like beads on a string (Fig. 2A and 2B). In 293 cells, which contain less endogeneous Cav-1 than NIH 3T3 cells [25] Cav-1-GFP as well as Gag-RFP were concentrated in patches at the edge of the cell, however, Cav-1-GFP accumulated predominantly in a perinuclear region in the center of the cell. Gag-RFP vesicles were also concentrated in this region, however, to a lesser extent (Data not shown). In cotransfected NIH 3T3 cells Gag-RFP and Cav-1-GFP spots co-localized to a certain extent at the PM and in perinuclear regions, but to a much lesser extent in the cytoplasm (Fig. 2A). Interestingly, when infected cells were used for the transfection experiments the Cav-1-GFP and Gag-RFP fluorescence patterns did not change considerably (see Additional file 1). However, in certain cases, Cav-1 predominantly stacked in perinuclear regions, while the pattern of Gag-RFP remained unaltered (data not shown).

In the experiments described endogenous Cav-1, even in low levels, may compete with Cav-1-GFP expression. Moreover, overexpression of Cav-1-GFP may favour stacking of the GFP fusion protein in perinuclear regions. Therefore, we repeated the experiments using Gag-RFP transfected cells and stained endogenous Cav-1 using an anti-Cav-1 antibody. Gag-RFP transfected NIH 3T3 cells were fixed 46 h after transfection and immunostained with rabbit anti-Cav-1 antibody and goat anti rabbit Alexa 488. Confocal optical sections taken through the cell every 0.5 μm revealed that Cav-1 as well as Gag-RFP are localized at the PM (see Additional file 2). Especially, Gag-RFP spots are scattered throughout the cytoplasm and perinuclear regions. However, both, Gag-RFP and Cav-1, co-localize in a dot-like pattern to a large extent at the PM (Fig. 2B, see Additional file 2). To improve visualization and to estimate the rate of co-localization 'correlation plots' were created. Therefore, the green and red channel were merged and co-localized pixels were highlighted in white. Approximately 40–70% of Gag-RFP and Cav-1 were co-localized at the PM (see Additional file 3). In addition, co-localization profiles revealed that, if not co-localized, Cav-1 and Gag spots at the PM are situated closely to another (see Additional file 4).

Taken together, our experiments reveal that Cav-1 and MLV Gag co-localize predominantly at the PM and to some degree in intracellular compartments. Moreover, MLV infection or the presence of other retroviral proteins does not influence the co-localization patterns.

### Gag-MA contains a putative caveolin-1 binding domain, which is highly conserved among γ-retroviruses

Cav-1 binds to a variety of cellular proteins via its caveolin scaffolding domain (CSD, aa82–101) [26]. Many of these binding partners play a role in cellular signaling. Two consensus domains for binding to Cav-1 (CBD) have been defined by phage display techniques using CSD as bait and a random peptide library [27]. Both consensus sequences were rich in aromatic residues and exhibited a characteristic spacing (ΦxxxxΦxxΦ; ΦxΦxxxxΦ; Φ = W, F, Y). Interestingly, we identified a putative CBD motif in the MA of MoMLV and A-MLV Gag precursors (Table 1) [19]. Strikingly, the motif is highly conserved within most γ-retroviruses (Table 1) and is absent in Gag of other retroviruses.

**Table 1:** Putative caveolin-1 binding domains in the matrix protein of the Gag precursor of γ -retroviruses

Retrovirus	AA*	protein sequence†	Accession No
Moloney MLV	31	KKRRWVT**F**CSAE**W**PT**F**NVGW	PIR:FOMV1M
Sp2/0 xenotropic retrovirus	31	KKRRWVT**F**CSAE**W**PT**F**GVGW	EMBL :X94150
Amphotropic MuLV 1313	31	KKRRWVT**F**CSAE**W**PT**F**NVGW	GenBank:AF411814.1
Abelson MLV	31	KKRRWVT**F**CSAE**W**PT**F**NVGW	Swiss-Prot:P03333
AKV MLV	31	KKRRWVT**F**CSAE**W**PT**F**NVGW	Swiss-Prot:P03336
Rauscher MLV	31	RKRRWVT**F**CSAE**W**PT**F**NVGW	GenBank:NP_044737
Friend MLV	31	RKRRWVT**F**CSAE**W**PT**F**NVGW	Swiss-Prot:P26805
MAIDS related virus (BXH-2)	31	RKRRWVT**F**CSAE**W**PT**F**NVGW	GenBank:AAB47858.1
MAIDS virus Duplan strain	31	KRRRWVT**F**CSVE**W**PS**F**NVGW	EMBL:X14576
Gibbon ape leukemia virus SEATO	31	KKGKWQT**F**CSSE**W**PT**F**GVGW	Swiss-Prot:21416
Gibbon ape leukemia virus X	31	RXGKWQT**F**CSSE**W**PT**F**GVRW	GenBank:AAC80263
Simian sarcoma virus	31	RKEKWQT**F**CSSE**W**PT**F**GVGW	PIR:FOMVGS
Moloney murine sarcoma virus	31	KKRRWVT**F**CSAE**W**PT**F**NVGW	PIR:FOMVM
Endogenous koala retrovirus	31	RKGKWQT**F**CSSE**W**PT**F**EVGW	GenBank:AAF15097
Porcine endogenous retrovirus	31	KKGPWQT**F**CASE**W**PT**F**DVGW	GenBank:CAB65341
*Mus dunni *endogeneous retrovirus (MDEV)	31	RKGPWQT**F**CASE**W**PT**F**GVGW	GenBank:AF053745
*Mus musculus *retrovirus (MmERV)	31	RKGPWQT**F**CTSE**W**PT**F**GVGW	GenBank:AC005743
Woolly monkey sarcoma virus	31	RKEKWQT**F**CSSE**W**PT**F**GVGW	Swiss-Prot :P03330

AA: Amino acid position of the first residue in the depicted sequences is indicated. Bold, CBD motif identified from consensus CBD motifs [27] which were rich in aromatic residues and exhibited a characteristic spacing (ΦxxxxΦxxΦ; ΦxΦxxxxΦ; Φ = W, F, Y).

### Caveolin-1 interacts with Gag precursor of MLV

**Figure 3:**
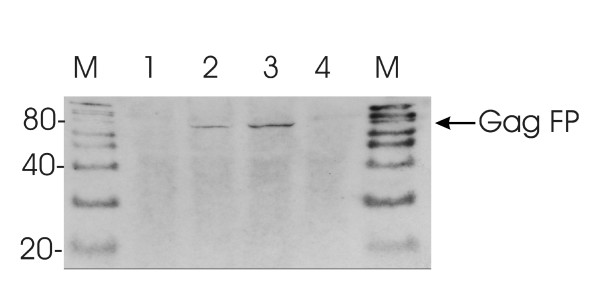
**Co-immunoprecipitation of Cav-1**. Lysates from or transfected (lane 1–3) or mock-transfected (lane 4) NIH3T3 cells were treated with rabbit anti Cav-1 followed by capture on paramagnetic protein G microbeads (μ column system, Miltenyi). Precipated proteins were separated by SDS-PAGE followed by Western Blot detection on PVDF membranes using an GFP antibody with GFP, YFP and CFP specifity. NIH3T3 lysates transfected with Lane1: GFP Plasmid; Lane 2: Gag-CFP; Lane 3: Gag-YFP Lane 4: mock; M Marker, molecular weight in kDa

Gag-Cav-1 co-localization, the presence of the putative CBD in MA of MLV and the high degree of conservation among γ-retroviruses motivated us to investigate whether the two proteins interact with each other. To determine whether MA-Cav-1 interactions occur in cells two types of binding experiments were carried out. In the first set co-immunoprecipitation experiments were performed using NIH 3T3 cells transfected with Gag-YFP or Gag-CFP expression plasmids. In these plasmids YFP or CFP variants have been fused to the C-terminus of MoMLV Gag [24]. Cells were lysed, Cav-1 was pulled down by an Cav-1 antibody/proteinG and the precipitates were separated by SDS PAGE (10%) and analyzed by Western Blot for their Gag-YFP or Gag-CFP content (Fig. 3). In samples transfected with Gag-CFP or Gag-YFP fusion plasmid (lane 2, lane 3) specific signals appeared at the expected size of 80 kD. No signals were detected in probes from mock-transfected NIH 3T3 cells (lane 4) or NIH 3T3 cells transfected with a GFP expression plasmid (lane 1), which excludes cross-reactions with GFP and Cav-1 during immunoprecipitation. Our data show that immunoprecipitation of Cav-1 results in recovery of a MLV Gag-Cav-1 complex and strongly indicates that Cav-1 binds to MLV Gag.

**Figure 4:**
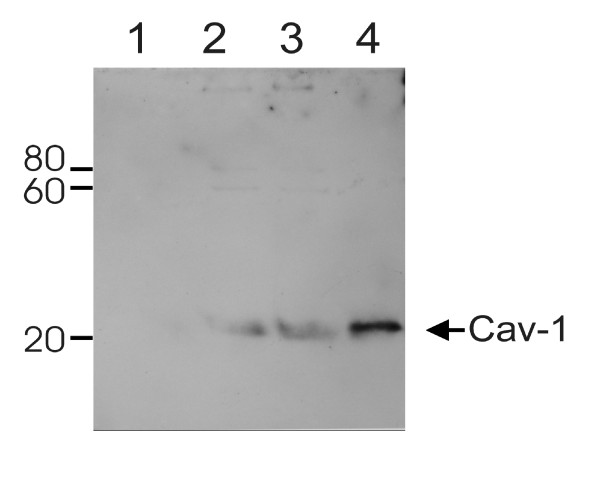
**Pull down experiments using CBD peptides**. 20 mM biotinylated peptide encompassing either the putative binding domain within MA or a consensus binding motif were inoculated with 50 μl NIH3T3 cell lysate for 90minutes (from 2 ml lysate of semiconfluent T75 culture flask). Complexes were immobilized using 10 μl streptavidin coated paramagnetic microbeads and μ column (Miltenyi). Washed samples were eluted and 15 μl of 80 μl eluate were separated by SDS-PAGE, blotted to PVDFmembrane and probed with anti-caveolin-1 antibody. lane 1: NIH 3T3 lysate, no peptide added ; lane 2 : NIH3T3 lysate with biotinylated CBD-MA peptide; lane 3: NIH 3T3 incubated with biotinylated consensus CBD peptide; lane 3: positive control, NIH3T3 lysate, non-processed. Molecular weights are depicted (kDa).

In another set of experiments biotinylated peptides were used as baits for binding partners. The synthetic peptides encompassed the putative CBD of MA (MoMLV) (KKRRWVT**F**CSAE**W**PT**F**NVGW-K-Biotin) or a consensus CBD (RNVPPI**F**NDVY**W**IA**F**NVGAR-K-Biotin) [27]. After incubation with the cell lysates, complexes were bound on paramagnetic streptavidin beads. The eluate was separated via SDS PAGE and Western Blots were probed with polyclonal anti-Cav-1 antibody (Figure 4). Signals co-migrating with the Cav-1 band of NIH 3T3 extracts (positive control) appeared when either the biotinylated CBD of MA or a consensus CBD were incubated with the extract, but no signal cold be detected from NIH3T3 extract alone. In addition, two signals of minor intensity could be detected at 60 kD and 80 kD which may represent oligomeric forms of Cav-1.

The experiments demonstrate that a synthetic peptide comprising the putative CBD of MA of MoMLV pulls down Cav-1 as efficient as a consensus CBD peptide defined by phage display [27]. Taken together both series of experiments provide compelling evidence that MLV Gag directly interacts with Cav-1.

### CBD expression interferes with virus production

**Figure 5:**
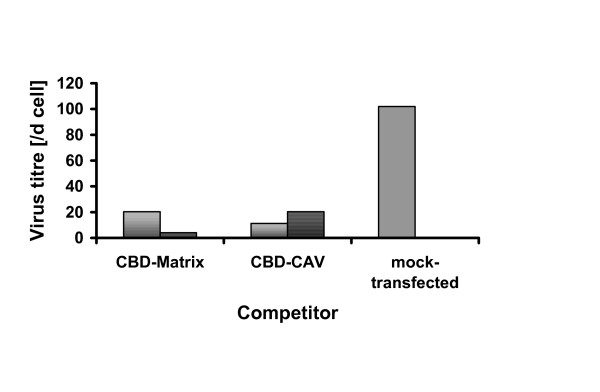
**Effect of CBD expression on MLV production**. Expression plasmids carrying cloned caveolin-1 binding domains were transfected into 4070A infected NIH3T3 cells and the effect on virus release was determined by infectious titer assay as described in Material and Methods. Competition experiments involved the putative CBD domain in MA of MLVs or a consensus CBD derived from display analysis [27].

We next performed experiments designed to investigate the biological significance of the Cav-1-Gag interaction. We reasoned that overexpression of fusion proteins containing the CBD of MA could block the interaction of Gag with endogenous Cav-1. To study the effect on virus formation expression plasmids were constructed encoding GFP fused to the CBD of MA or the consensus CBD peptide [27] as a positive control. Expression plasmids were transiently transfected into A-MLV producing NIH3T3 cells and the effect of CBD overexpression on infective virus production was determined by infectious titre assay (Figure 5). Results of three experiments show that virus production was reduced 5–10 fold upon CBD transfection. This suggests, that CBD overexpresssion competes with endogenous Cav-1 for binding to Gag and that Cav-1 indeed plays a functional role in A-MLV production.

### A dominant negative caveolin-1 mutant down-modulates virus production

**Figure 6:**
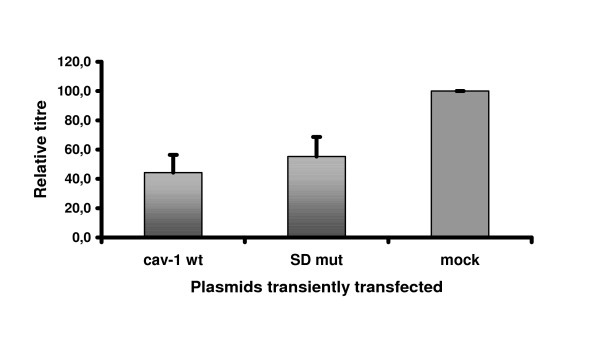
**Influence of expression of cav-1 wild-type and dominant-negative mutant cav-1 on MLV-A titres in NIH3T3 cells**. MLV-A infected NIH3T3 cells were transiently transfected with expression plasmids containing wt cav-1 cDNA or a dominant-negative mutant carrying to mutations in the scaffolding domain [28]. Titres were determined from supernatants 48 h after transfection on indicator cells according to Spearman and Karber as described in Materials and Methods. Normalized values are shown. In each of three independent experiments mock-transfected NIH-MLV-A were used for normalization. Standard deviations are shown.

If endogenous Cav-1 is important for A-MLV production overexpression of Cav-1 or the interaction with a scaffolding-incompetent Cav-1 mutant should exert similar, negative effects on virus yield. To evaluate the role of Cav-1 the effect of overexpressing wild-type (wt) Cav-1 or the dominant-negative Cav-1 mutant [28] on A-MLV virus production was investigated in infected NIH 3T3 cells which are known for their high Cav-1 content. Therefore, wt Cav-1 or Cav-1 Mut SD expression plasmid were transiently transfected into NIH 3T3 cells releasing a G418 resistant A-MLV and 48 hours after transfection viral titres were determined on indicator cells. A significant reduction in virus titre was observed in Cav-1 Mut SD transfected cells when compared to mock-transfected cells (Figure 6). Interestingly, NIH 3T3 cells transfected with the wt Cav-1 construct exhibited a similar reduction in viral titre. These experiments suggest that inhibition of Cav-1 function as well as overexpression interfere with virus production and point to a discrete role of Cav-1 in late viral processes.

## Discussion

We presented evidence that MLV Gag interacts with Cav-1 and that this interaction influences virus assembly and production. As another consequence of its interaction with Gag, Cav-1 is incorporated into A-MLV and MoMLV released from NIH 3T3 cells. Confocal fluorescence microscopy revealed that Cav-1 and MLV Gag co-localize predominantly in punctate patterns at the PM and to lower extent in perinuclear regions of the cell. Sequence comparisons uncovered a Cav-1 binding domain in the matrix domain MA of the Gag precursor which is highly conserved among γ-retroviruses. Subsequent binding experiments using co-immunoprecipitation and a pull-down assay revealed that Cav-1 directly interacts with MLV-Gag. The interaction of Cav-1 with MA seems to play an important role in virus production, as overexpression of the CBD of MA considerably reduced the production of infectious virus in NIH 3T3 cells. Furthermore, overexpression of both, wt Cav-1 and a dominant-negative Cav-1 mutant, in A-MLV releasing NIH 3T3 cells resulted in a considerable decrease of virus production.

### The role of Cav-1 in the MLV life cycle

There are several lines of evidence that Cav-1 incorporation into virus and its interaction with Gag is of biological relevance. First, the CBD is highly conserved within γ-retroviruses. The strong selective pressure on preservation of the sequence argues for performance of a specific function within the viral life cycle. Second, perturbing the stochiometry of the interaction between Gag and Cav-1 by expression of wt Cav-1, a dominant negative Cav-1 mutant or fusion proteins carrying the CBD impaired viral life cycle and resulted in considerable decrease in viral yield. The inhibition is a specific process rather than an effect exhibited by interfering with cell viability or physiology. Obviously, perturbation interferes with late processes in viral replication. Third, further hints for the importance of the CBD of MA arise from deletion or linker scanning mutational analysis of the MA protein function performed earlier [5,29-31]. Strikingly, deletion or linker scanning mutation encompassing the region of the putative CBD resulted in dislocation of Gag and decrease in virus yield. For example, mutation of the tryptophan residues in a 'hydrophobic region', especially those which are part of (W43) or are close (W35, W50) to the putative CBD of MLV Gag (amino acids 38–46 of the Gag precursor) resulted in a dramatic loss in the production of infectious MoMLV and decrease in viral reverse transcriptase (RT) activity of released viruses. Mutations in residues 40, 44, 45 and 46 resulted in a 20 fold decrease in viral infectivity compared to the wt virus. As in this analysis the Gag localization pattern of tryptophan mutants differs considerably from wt Gag – the mutant Gag localizes exclusively in perinuclear regions in diffuse manner – the CBD tryptophan residues in MA identified in our investigation have been suggested to play an important role in Gag transport [5].

### Positioning of Gag to cellular membranes

It is conceivable that Cav-1-Gag interaction is crucial for positioning MLV Gag at the PM, special PM domains like lipid rafts and/or the membrane of intracellular compartments or vesicles. If Cav-1 functions in that way it would function as a Gag receptor. The existence of a Gag receptor has been postulated, since the membrane insertion reaction is highly efficient and specific [32]. Retroviral Gag precursor proteins become anchored into the cytoplasmic leaflet of the PM via a dual motif consisting of amino terminal myristoylation and a cluster of basic residues [2-6,8]. The dual motif is not expected to result in a very specific insertion, as myristoylated proteins are found in several compartments and acidic phospholipids, which interact with basic amino acids, are not restricted to the PM.

Interestingly, Cav-1 interaction with proteins substitutes for fatty acylation in certain cases and has been described to help in localization of proteins to lipid rafts. Partitioning of acyl side chains into liquid-ordered phase domains has been suggested as mechanism for targeting of proteins to lipid rafts [33]. However, although fatty acylation is necessary for membrane association of proteins in general, there is certain evidence that the normal requirement for acylation for localization in lipid rafts can be overcome by other molecules [34]. Studies with acyl-modified GFPs showed that N-terminal protein acylation only conferred localization to cholesterol and sphingolipid-enriched membranes but not to lipid rafts or caveolae, suggesting that protein-protein interactions may be required for efficient raft association [34]. Also, acylated vesicular stomatitis virus (VSV) G protein and Rous sarcoma virus (RSV) Env were not associated with lipid raft [35]. Interestingly, it has been shown, that overexpression of a recombinant caveolin in intact cells is sufficient to functionally recruit a non-farnesylated Ras mutant onto membranes thereby overcoming the normal requirement for lipid modification of Ras. This suggests that caveolin may function as scaffolding protein to localize or sequester caveolin interacting proteins (e.g. wt Ras) within caveolin-rich microdomains of the PM [34]. Interestingly, caveolin is palmitoylated at 3 residues, but fatty acylation is not necessary for its caveolae localization [36]. Moreover, Pr60 Gag of murine AIDS virus lacking the myristoyl modification is not dispersed in the cytoplasma like MoMLV Pr65 Gag, but attaches loosely to the PM [37]. Overexpression of caveolin-1 in cells infected with a myristoylation minus MLV mutant and analysis of Gag localization and transport of mutants encompassing the MA-CBD motif and neighbourhood will elucidate more details on the importance of caveolin in Gag membrane attachment.

Presently, we do not know how many functions Cav-1 exerts in the MLV replication cycle. However, our results suggest that Cav-1 presumably is responsible for Gag localization within lipid rafts. According to our present understanding Cav-1 containing lipid rafts rather than caveolae itself seem to be most suitable for assembly and budding, as invagination, endocytosis and the compact coat of caveolae would exclude virus budding. Such an interpretation is supported by the characteristic localization patterns exhibited in profile analysis where co-localization and to some extent nearby localization of Gag and Cav-1 at the PM could be observed and a release process may be assumed when caveolae are formed upon Cav-1 oligomerization from preformed multimers. Furthermore, once localized, binding to Cav-1 may initiate oligomerization of further Gag molecules, leading to Gag clustering, a crucial oligomerization step in virus formation. Possibly, Cav-1 may also play a role in the transport of Gag to intracellular vesicles like MVBs or to the PM, either membrane bound, in its soluble form or asscociated with lipid droplets [38]. Finally, due to the fact that Cav-1 co-localizes with MLV-Env and Gag, it may serve as a Gag-Env bridging molecule. However, unlike in RSV or HIV, there is little evidence for such a close linkage in the case of MLV [39,40].

Taken together, it is likely that Cav-1 functions to locate MLV Gag to the PM, and due to the co-localization of Env, Cav-1 and GM1 [16], a marker for lipid rafts, a role for Cav-1 in Gag positioning in lipid rafts is highly probable. It is tantalizing to speculate, that this will also hold for Gag of related retroviruses listed in Table 1 which contain the CBD motif.

Recently, Hovanessian et al. reported that HIV-gp41, the transmembrane subunit of the viral spike protein, also binds to caveolin-1 via a CBD motif located at position 622–633 of gp41 [41]. The CBD in gp41 is highly conserved within HIV-isolates and SIV lentivirus. However, the binding region was mapped to the lentiviral ectodomain of the transmembrane protein and the function of this putative interaction has not been revealed. Due to the external location of the CBD Cav-1 incorporation into virions has not been observed [41].

## Conclusion

Taken together our data demonstrate that Cav-1 co-localizes with Gag of murine leukemia viruses at the PM and interacts with this precursor protein via a CBD in MA. As CBD competition or overexpression of a dominant-negative Cav-1 mutant affects virus production, Cav-1 plays distinct roles in virus assembly.

## Methods

### Cells and viruses, cell culture

NIH 3T3 cells (ATCC CRL-1658) and 293 cells (ATCC CRL 1573) were propagated in DMEM supplemented with antibiotics, glutamine and 10% FCS. Cells were grown at 37°C, 5% CO_2 _and 95% humidity.

### Plasmids, transfections and helper virus approach

pMLV ampho and pMLVeco contain the complete genome of amphotropic MLV or ecotropic MLV cloned into pBluescript (Genethon, France, received via J.-C. Pages). pCaveolin-1-GFP contains canine cav-1 cDNA followed in frame by EGFP [21]. pCFPgag and pYFPgag are in frame fusion of MoMLV Gag with CFP or YFP [24]. pMLVgagRFP contains the MoMLV Gag ORF inserted into the BamHI/AgeI site of pmRFP-N1 (R.Tsien)[23]. pCSD-MLV and pCSD-Consensus were created by insertion of an oligonucleotide coding for the putatitive CBD domain in MA and a oligonucleotide coding for the consensus CBD (see peptides), respectively, into the EcoRI site of pTarget (Promega). pLEIN contains a bicistronic MLV vector harboring EGFP and the neonmycin resistance gene (Clontech). pCav-WT contains a myc-tagged canine Cav-1 cDNA copy cloned into pCIS2 [28]. pCav-MUT contains point mutations (F92A V94A) in the scaffolding domain of canine cav-1 cDNA [28]. Transfections were performed using purified DNA (Quiagen kit) and the calcium-phosphate coprecitation method. MLV producing NIH3T3 cells resulted from calcium phosphate transfection of pMLVampho or pMLV eco, respectively, and subsequent infection of NIH3T3 with the respective replication competent MLV. G418 resistant viruses were created by cotransfection of pMLVs with pLEIN, which contains a bicistronic MLV vector harboring EGFP and the neomycin resistance gene (Clontech).

### Virus isolation

MLVs were precipitated from cleared supernatants of MLV infected NIH 3T3 cells from three T75 flasks (2000 rpm, Heraeus Megafuge 1R) by centrifugation (3 h, 17000 rpm Sorvall FAD-20C). Virus pellets were resuspended in TNE and purified by sucrose gradient centrifugation (25–40% discontinuous, O/N 35 000 rpm). Viruses banding at approx. 35% sucrose were collected and precipitated at 40000 rpm for 3 h. All steps were carried out at 4°C. Virus pellets were resuspended in 100 μl TNE and stored at -20°C.

### Virus titration

Virus mediating G418 resistance were created by the helper approach and titrated on NIH 3T3 indicator cells (750 c per well, microtiter plate 96 well, 8fold determination) according to the method of Spearman and Kaerber [42]. Serial dilutions of filtered supernatants (24 h production) were prepared and infection of indicator cells was performed in the presence of 8 μg/ml polybrene. Selective medium was applied 2d after infection and clone forming units (cfu) were determined 10 d after infection by staining of cells with crystal violet.

### Peptide synthesis

Biotin-labelled CBD peptides were synthesized by the group of Werner Tegge (Chemical Biology, GBF, Braunschweig, Germany). CBD-MA contained the putative CBD binding domain in MA (sequence AcRNVPPIFNDVYWIAFNVGAR-K-Biotin), CDB consensus a consensus Cav-1 binding domain deviated from phage display experiments (sequence AcKKRWVTFCSAEWPTFNVGW-K-Biotin) [27].

### Lysis of cells and viruses

Cells or viruses were treated with lysis buffer (10 mM Tris pH7.5, 50 mM NaCl, 1% Triton X100, 60 mM octylglucoside (Roche), 1 mM aprotinin, 1 mM leupeptin, 1 mM PMSF) at 4°C for 30 min followed by centrifugation in an Eppendorf centrifuge at 15000 rpm. Cleared supernatants were processed further or stored at -20°C.

### Protein/protein binding assays

#### Co-immunoprecipitation

Cell lysates were incubated with rabbit anti-caveolin-1 antibody (1:2000) at 4°C for 1 h followed by incubation with 50 μl Protein G-microbeads at 4°C for 1 h and subsequent application to prewashed μ columns (Miltenyi). After four washing steps (200 μl lysis buffer) bound proteins were eluted with 70 μl sample buffer preheated to 95°C.

#### Pull-down assays

Cell lysates were incubated with 20 μM biotinylated CBD motif peptides at 4°C for 90 min followed by treatment with 10 ml streptavidin coated microbeads (Miltenyi Biotec). Lysate was applied on prewashed μ columns and after washing five times with 200 μl lysis buffer the proteins were eluted with 70 μl sample buffer preheated to 95°C.

### SDS-PAGE and Western Blot analysis

Proteins were separated by SDS-PAGE (12%) at 100 V for 2 h. Semidry blotting was used for subsequent protein transfer to PVDF membranes. After O/N blocking with Starting Block (PerBio), primary antibody (rabbit anti-caveolin-1 or mouse anti-GFP diluted 1:2000 in starting block buffer containing 0.05% Tween20) was applied at room temperature with constant shaking for 1 h. Membranes were washed 3 times for 10 min in TBS/0.05%Tween20 followed by incubation with the secondary antibody (goat anti-rabbit HRP 1:100,000 or goat-anti mouse HRP 1:1,000,000). After 5 additional washes membranes were incubated in luminal/enhancer solution (PerBio).

### Immunostaining and confocal immunofluorescence

Cells were either fixed with 4% formaldehyde (cotransfections of fluorescent fusion proteins) or fixed/permeabilized with cold methanol/aceton (Gag-RFP, endogenous Cav-1) on coverslips. Blocking (2% goat serum in PBS for 20 min) was followed by incubation with the primary rabbit anti-caveolin-1 antibody (Transduction Laboratories, 1:300 diluted in PBS/2% goat serum) for 1 h. Excess antibody was removed by washing three times with PBS containing 0.02% TritonX-100. To detect the primary antibody, the samples were incubated with an Alexa Fluor 488-labeled goat-anti-rabbit secondary antibody (Molecular Probes, 1:300 dilution, Alexa Fluor 488 F(ab')_2 _conjugate IgG (H+L)). The coverslips were washed again and then mounted onto glass slides using fluorescent mounting medium (DAKO). Confocal imaging was performed with a Zeiss LSM 510META laser scanning microscope (inverted Axiovert 200 M microscope) using a Plan-Apochromat 100× oil immersion objective (1.3 numeric apertures). EGFP or Alexa Fluor 488-labelled antigens were excited with an argon laser at 488 nm, and emission was monitored using a 505–530 nm bandpass filter. For RFP visualization a HeNe laser at 543 nm and a 560–615 nm bandpass filter were used.

### Competition and inhibition experiments

Plasmids pCSD-consensus or pCSD-MLV were stably introduced into A-MLV/pLEIN infected NIH3T3 cells by calcium phosphate precipitation. Virus titers were determined from pooled clones.

Plasmids pCav-wt or pCav-Mut were transiently introduced into A-MLV/pLEIN infected NIH3T3 cells by calcium phosphate precipitation. Virus titers were determined 2 d after transfection.

## Abbreviations

CBD, caveolin binding domain; Cav-1, caveolin-1; MoMLV, murine leukaemia virus; A-MLV, amphotropic murine leukaemia virus; PM, plasma membrane; Env, envelope protein; Gag group-specific antigen; HIV, human immunodeficiency virus; GFP green fluorescent protein; YFP, yellow fluorescent protein; CFP, cyan fluorescent protein; RFP, red fluorescent protein; PMSF, phenyl methyl sulfonyl fluoride;

## Competing interests

The author(s) declare that they have no competing interests.

## Authors' contributions

MW and CB conceived the study. CB and MW performed the competition experiments. CB and ZY studied incorporation of cellular protein into virions. ZY performed the co-immunoprecipitations, pull down experiments and co-localization experiments. MK trained ZY in confocal microscopy and provided input for the fluorescenct colocalization experiments. MW performed the inhibition experiments, supervised all the experiments and drafted the manuscript. All authors read and approved the final manuscript.

## Supplementary Material

Additional File 1 **Colocalization of Cav-1 and Gag RFP in transfected A-MLV infected NIH3T3 cells. **A-MLV infected NIH3T3 were transfected with GagRFP plasmid, fixed 46 h after transfection and stained for immunofluorescence rabbit anti-caveolin-1antibody followed by goat anti-rabbit-Alexa 488 conjugate. Click here for file

Additional File 2 **Colocalization of Cav-1 and Gag RFP in transfected NIH3T3. Z-Stack images**. NIH3T3 transfected with GagRFP plasmid were fixed 46 h after transfection and stained for immunofluorescence rabbit anti-caveolin-1antibody followed by goat anti-rabbit-Alexa 488 conjugate. Scanning by confocal microscopy from bottom to top, distance or 0.5 μm each. Click here for file

Additional File 3 **Correlation plot and colocalization points of Cav-1 and Gag RFP fluorescence in NIH3T3 cells**. The software merges the red (Ch3-T2) and green channel (Ch2-T1) and highlights colocalized pixels in white. Pixels are considered colocalized when their intensity is higher than the threshold of their channels (red label), which was defined by analysing the distribution frequency. Click here for file

Additional File 4 **Profile analysis of Cav-1 and Gag RFP fluorescence in NIH3T3 cells**. Profile was drawn by Zeiss software and depicts the intensity distribution (B) in the channels detecting GagRFP (red) and caveolin-1 (green) along the red arrow (A). Click here for file
