# Supplementary figures and images for: Caveolin-1 interacts with the Gag precursor of murine leukaemia virus and modulates virus production
Source: Virol J. 2006 Sep 6;3:73. doi: 10.1186/1743-422X-3-73 (PMC1570462; doi:10.1186/1743-422X-3-73)

### Additional file 1

###
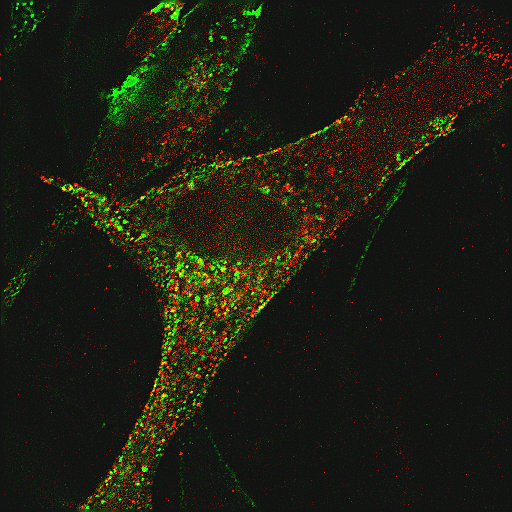

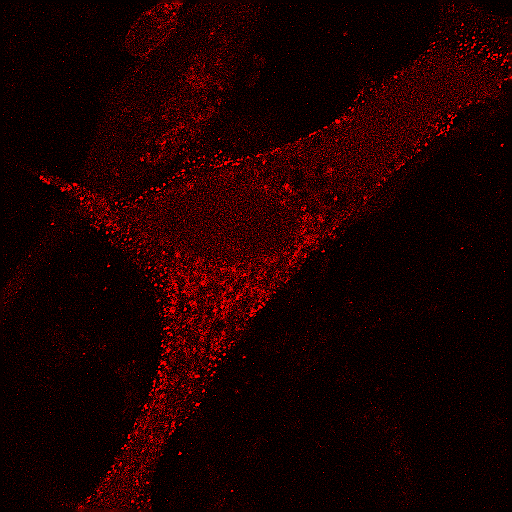

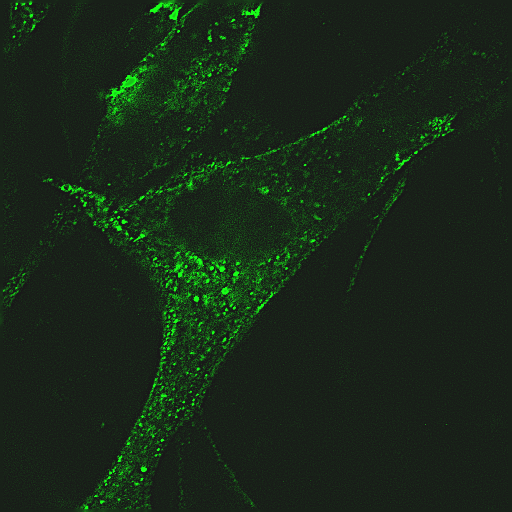


10 µm

**GagRFP Cav-1**

**Merged**

Supplement: Additional File 1 — Colocalization of Cav-1 and Gag RFP in transfected A-MLV infected NIH3T3 cells. A-MLV infected NIH3T3 were transfected with GagRFP plasmid, fixed 46 h after transfection and stained for immunofluorescence rabbit anti-caveolin-1antibody followed by goat anti-rabbit-Alexa 488 conjugate. [file 1743-422X-3-73-S1.doc]

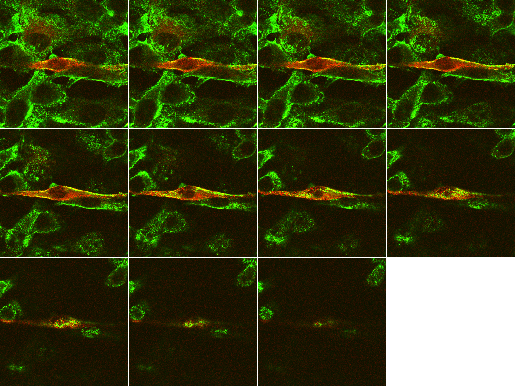

Supplement: Additional File 2 — Colocalization of Cav-1 and Gag RFP in transfected NIH3T3. Z-Stack images. NIH3T3 transfected with GagRFP plasmid were fixed 46 h after transfection and stained for immunofluorescence rabbit anti-caveolin-1antibody followed by goat anti-rabbit-Alexa 488 conjugate. Scanning by confocal microscopy from bottom to top, distance or 0.5 μm each. [file 1743-422X-3-73-S2.doc]

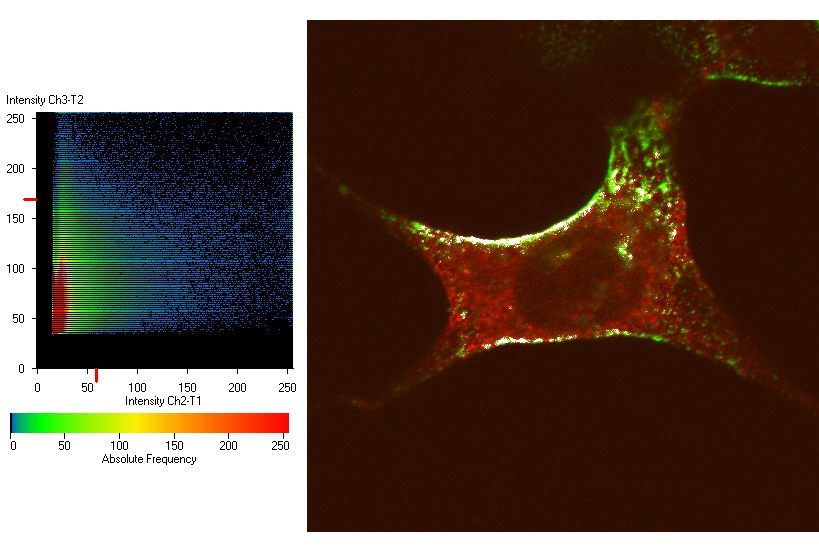

Supplement: Additional File 3 — Correlation plot and colocalization points of Cav-1 and Gag RFP fluorescence in NIH3T3 cells. The software merges the red (Ch3-T2) and green channel (Ch2-T1) and highlights colocalized pixels in white. Pixels are considered colocalized when their intensity is higher than the threshold of their channels (red label), which was defined by analysing the distribution frequency. [file 1743-422X-3-73-S3.doc]

**
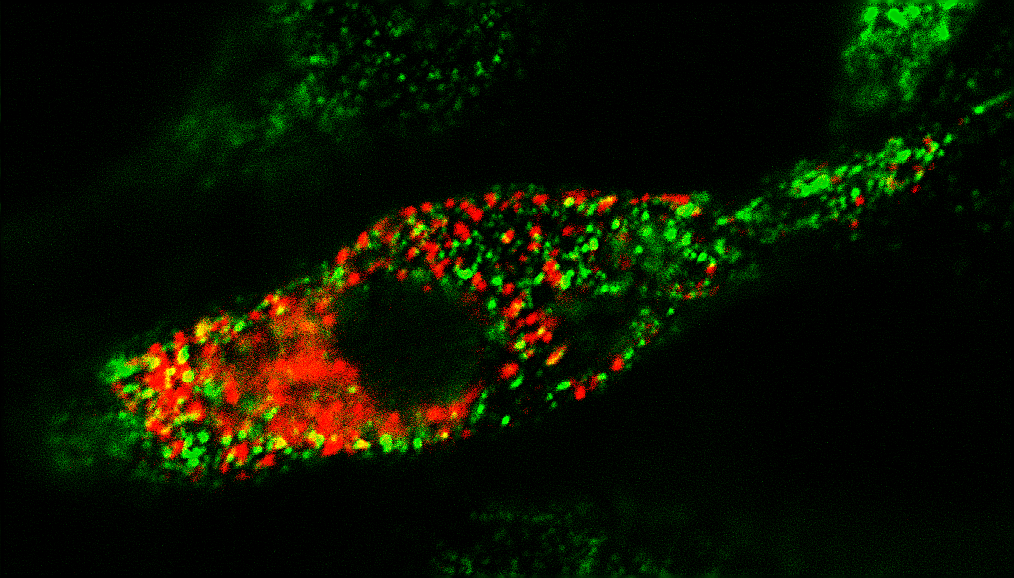
**

Supplement: Additional File 4 — Profile analysis of Cav-1 and Gag RFP fluorescence in NIH3T3 cells. Profile was drawn by Zeiss software and depicts the intensity distribution (B) in the channels detecting GagRFP (red) and caveolin-1 (green) along the red arrow (A). [file 1743-422X-3-73-S4.doc]
